# Regional Failures after Selective Neck Dissection in Previously Untreated Squamous Cell Carcinoma of Oral Cavity

**DOI:** 10.1155/2014/205715

**Published:** 2014-03-11

**Authors:** Hassan Iqbal, Abu Bakar Hafeez Bhatti, Raza Hussain, Arif Jamshed

**Affiliations:** ^1^Department of Surgical Oncology, Shaukat Khanum Memorial Cancer Hospital and Research Centre, 7A Block R-3, M.A.** **Johar Town, Lahore, Pakistan; ^2^Department of Radiation Oncology, Shaukat Khanum Memorial Cancer Hospital and Research Centre, 7A Block R-3, M.A.** **Johar Town, Lahore, Pakistan

## Abstract

*Aim*. To share experience with regional failures after selective neck dissection in both node negative and positive previously untreated patients diagnosed with squamous cell carcinoma of the oral cavity. *Patients and Methods*. Data of 219 patients who underwent SND at Shaukat Khanum Cancer Hospital from 2003 to 2010 were retrospectively reviewed. Patient characteristics, treatment modalities, and regional failures were assessed. Expected 5-year regional control was calculated and prognostic factors were determined. *Results*. Median follow-up was 29 (9–109) months. Common sites were anterior tongue in 159 and buccal mucosa in 22 patients. Pathological nodal stage was N0 in 114, N1 in 32, N2b in 67, and N2c in 5 patients. Fourteen (6%) patients failed in clinically node negative neck while 8 (4%) failed in clinically node positive patients. Out of 22 total regional failures, primary tumor origin was from tongue in 16 (73%) patients. Expected 5-year regional control was 95% and 81% for N0 and N+ disease, respectively (*P* < 0.0001). Only 13% patients with well differentiated, T1 tumors in cN0 neck were pathologically node positive. *Conclusions*. Selective neck dissection yields acceptable results for regional management of oral squamous cell carcinoma. Wait and see policy may be effective in a selected subgroup of patients.

## 1. Introduction

Since description of neck dissection in late 19th century, modifications have been proposed, practiced, and argued. Tracing back the heritage of neck dissection, sequential evolution from a morbid to a cosmetically tailored and oncologically acceptable procedure becomes evident. Although several different classifications have been adapted in the past, debate on a balanced and widely acceptable nomenclature continues. Lately selective neck dissection (SND) has been the buzz word for regional management in head and neck cancer. Shah [1] demonstrated frequency and patterns of regional lymph node metastases from oral squamous cell carcinoma in patients who underwent radical neck dissection. By definition SND refers to preservation of 1 or more lymph node levels. Although SND is an accepted procedure for pathological staging of clinically node negative (cN0) neck, the house remains divided between elective neck dissection versus a more conservative wait and see policy [2]. The therapeutic role of SND in clinically node positive (cN+) disease is still unclear but is gaining popularity in carefully selected patients [3, 4]. The exact protocol for regional management of squamous cell carcinoma of oral cavity is yet to be established. The objective of present study was to report regional control with selective neck dissection in both N0 and N+ previously untreated patients diagnosed with squamous cell carcinoma of the oral cavity.

## 2. Methods

A review of patients who underwent SND between 2003 and 2010 at Shaukat Khanum Memorial Cancer Hospital and Research Center was performed. A total of 219 patients who underwent SND for histology proven squamous cell carcinoma (SCCa) of oral cavity during the study period were included. Patients who received any treatment elsewhere were excluded from the study. All patients underwent a comprehensive clinical examination of head and neck followed by MRI of the face and neck and chest X-ray. Patients were staged according to the AJCC (American Joint Commission on Cancer) guidelines. The management protocol of these patients was tailored in the weekly multidisciplinary team clinic. SND was the mainstay surgical protocol for regional control alongside wide local excision of tumor. For patients diagnosed with SCCa of upper alveolus, maxillectomy was performed and SND was reserved for patients with clinically or radiologically node positive disease. Inclusion criteria for performing SND included tumor >1.5 cm and tumor thickness of more than 4 mm. Adjuvant treatment options such as postoperative radiotherapy (PORT) or concurrent chemoradiotherapy (CRT) were planned for the patients after pathological staging.

## 3. Induction Chemotherapy

Main indication for induction chemotherapy (IC) was bulky local and inoperable disease. Other indications included patients with tumors crossing midline, involvement of tip of tongue, and extension into the base of tongue or floor of the mouth. A total of 45 patients received IC prior to surgery. Induction chemotherapy was administered on outpatient basis. The regimen comprised of a combination of 2 drugs: intravenous gemcitabine 1000 mg/m^2^ on day 1 and day 8 and cisplatin 75 mg/m^2^ on day 1 of each cycle, respectively. A 3-week interval was observed between the 2 cycles. After 2 weeks from the second cycle, a response assessment was clinically devised and patients were planned for wide local excision of the tumor along with SND.

## 4. Surgical Management

Wide local excision was performed in tongue, lips, retromolar trigone, and buccal mucosa with 1 cm clear margin. Patients with squamous cell carcinoma of lower alveolar mucosa underwent marginal or segmental mandibulectomy depending on extent of involvement of lower alveolus. Maxillectomy was performed for patients with SCCa of upper alveolus. Frozen section was reserved for patients with clinically suspicious mucosal tissue. Majority of patients with T1 and early T2 tumors (<3 cm) and cN0 underwent SND I–III. Patients with advanced T2 (>3 cm), T3, and T4 tumors or clinically N+ disease underwent SND I–IV. After extraction of neck specimen, sublevels were separated and placed individually in formalin filled containers and sent for histopathological analysis. A template was prepared by pathologist to interpret the report including the number of nodes harvested, size of the fibro fatty tissue, and number of positive nodes. The presence of perineural invasion, lymphovascular invasion, and extra capsular spread was also documented for each level of neck specimen. Bilateral neck dissection was performed in patients with radiological evidence of contralateral neck disease.

## 5. Adjuvant Treatment

Postoperative radiotherapy (PORT) was used in patients with pathologically node positive disease, >1 cm tumor size, >5 mm tumor thickness, and poorly differentiated tumors. In pathologically node negative patients, 60 Gy in 30 fractions was given to the primary site and ipsilateral neck. In pathologically node positive patients, 60 Gy in 30 fractions was given to the primary site and bilateral neck. Concurrent chemoradiotherapy (CRT) was reserved for patients with extra capsular spread, perineural invasion, lymphovascular invasion, and more than 2 positive lymph nodes.

## 6. Statistical Analysis

Patient characteristics and treatment modalities were observed. Patients who did not have contralateral neck dissection were not included as regional failures. Regional control was calculated by subtracting date of failure from date of surgery. Expected 5-year regional control was calculated using Kaplan-Meier curves and significance between variables was determined with log rank test. Statistical Package for Social Sciences (SPSS), version 17, was used for statistical analysis.

## 7. Results

### 7.1. Patient Characteristics

A total of 219 patients underwent SND of which 158 were clinically node negative and 61 were node positive. Median age at presentation for node negative patients was 51 (13–76) years and median follow-up was 2.9 (0.07–9) years. Median age at presentation for node positive patients was 50 (28–79) years and median follow-up was 1.8 (0.2–7) years. Anterior tongue was the most common subsite with nearly 72% patients. Majority of patients received postoperative radiotherapy. Forty-five patients received induction chemotherapy.  Table 1 summarizes patient characteristics and treatment modalities with respect to clinically node negative and positive patients. There was a significant difference between two groups with respect to site of primary tumor (*P* = 0.009), treatment received (*P* = 0.0001), and sublevels dissected (*P* = 0.002). Anterior tongue was the site of primary in 75% patients with N0 neck disease versus 66% with N+ neck disease. Surgery alone was the treatment modality in 16% patients with clinically N0 disease and 2% patients with N+ disease. Sublevels I–III were dissected in 21% patients with N0 versus 3% patients with N+ disease. Out of a total of 52 patients with unknown extracapsular status, 65% patients received postoperative radiation, and 25% received concurrent chemoradiotherapy based on presence of other poor prognostic factors. Postoperative radiotherapy was used in 193 (88%) patients. Out of these, 51 (26%) had concurrent chemoradiotherapy.

#### 7.1.1. Clinical and Pathological Stage

Table 2 represents the clinical and pathological distribution of the study cohort. Locally advanced (T3/T4) tumors were found in 26% patients. One hundred and fifty-eight (72%) patients were cN0 at the time of presentation. One hundred and twenty-one (55%) patients had advanced disease (stage III/IV) on histopathology. Mean number of extracted nodes was 50 nodes and a total of 11936 nodes (level 1, 1836; level 2a, 2500; level 2b, 1600; level 3, 3000; level 4; 3000) were extracted in 219 patients. Occult nodal disease was present in 58 (37%) patients. Level II A was the most commonly involved sublevel in tumors of anterior tongue while level I was most frequently involved in tumors of buccal mucosa and lower alveolus.

### 7.2. New Classification

In Table 3, the newly proposed classification of neck dissection was compared with older version. None of the patients who underwent level I–III neck dissection had removal of any nonlymphatic tissues. Thirty-three patients out of 184 who underwent level I–IV SND had one or more nonlymphatic structures removed and were clearly demonstrable in new classification. Out of these 33 patients, 4 (12%) patients had extra capsular spread on histopathology (IJV = 3, IJV+SAN = 1). Rest had nonlymphatic structures removed due to perinodal fibrous adhesions.

### 7.3. Induction Chemotherapy

A total of 45 patients received induction chemotherapy. Male to female ratio was 2 : 1.  Table 4 represents their characteristics. On clinical exam, 65% patients had locally advanced (T3/T4) tumors. There was 1 patient with a T1 tumor on clinical exam who received induction. This patient had squamous cell carcinoma of tongue crossing midline. Histopathology of resected specimen after induction chemotherapy demonstrated that only 7% patients had T3/T4 tumors. Complete pathological response was seen in 6 patients. This difference was not observed for nodal involvement after induction chemotherapy. The expected 5-year overall survival for patients who received induction chemotherapy versus those who did not was 69 and 74%, respectively, and was not significantly different (*P* = 0.4).

### 7.4. Regional Failures in Clinically Node Negative and Node Positive Patients

Table 5 demonstrates failures in clinically node negative and positive patients. Total number of regional failures was 22. Fourteen (8.8%) patients failed in clinically node negative neck while 8 (13%) failed in clinically node positive patients. The most common tumor size stage was T2 in 45% patients. Tongue was the most common site of primary in 16 (72%) patients. Out of total 7 patients with extracapsular extension, 3 (42%) patients developed regional failure. Median recurrence-free survival in pN+ patients with and without extracapsular spread was 1.6 (0.08–9) and 1.7 (0.02–7) years and was not significantly different. A total of 14 patients had ipsilateral failures, including 3 patients that failed both locally and regionally. All patients that failed ipsilaterally underwent SND I–IV. Almost all patients that failed ipsilaterally in the neck were either treated with palliative chemotherapy or symptomatically. A total of 4 patients failed contralaterally in the neck.

There were 38 local, 19 regional, 3 locoregional, and 6 distant failures (not shown). None of the patients who underwent SND I–III failed ipsilaterally. Eight patients had occult metastatic disease after SND I–III, that is, 3 patients in level III, 5 in level II, and 3 in level I. Two patients underwent bilateral SND I–III as they were staged radiologically N2c; on histopathology they had N1 and N2b disease, respectively. Both these patients had SCCa of the anterior tongue. One patient had skip metastasis with pathologically positive node in level III, escaping levels I and II. Level II B dissection was performed in all patients. A total of 184 patients underwent SND I–IV of which thirteen patients had bilateral neck dissection. Ten patients showed skip metastasis of which nine patients had SCCa oral tongue and one had SCCa buccal mucosa. Level II B was removed in all patients. A total of 14 (7.6%) patients had positive lymph nodes in level IV but no isolated level IV involvement was seen. Also, 12/14 patients with level IV involvement had more than two positive nodes. Five of the fourteen patients had cN0 disease at presentation and on staging MRI ten patients had radiologically significant nodal disease.

### 7.5. Prognostic Factors

Grade, lymphovascular invasion, and pathological N stage were statistically significant for 5-year regional control (Table 6). A highly significant difference in regional control was present between N0 and N+ patients with expected 5-year control of 95% and 81%, respectively (*P* = 0.005). The 5-year overall survival and disease-free survival for the whole group were 73% and 61%, respectively (not shown).

Table 7 represents the rates of pathological nodal positivity after SND in patients with well differentiated tumors and clinically node negative neck. Almost 90% patients with T1 tumors in this subgroup had pN0 disease.

## 8. Discussion

Regional spread of oral cancer continues to be the most significant prognostic factor and decreases survival by 50% [5, 6]. A better understanding of lymphatic spread has shown that lymph drains within aponeurotic compartments [7]. Studies have shown that lymphatic spread with respect to anatomical subsite can be predicted [8, 9]. Shah [1] mapped the lymphatic spread for squamous cell carcinoma of upper aerodigestive tract in 501 patients with oral cancer. Studies have shown 0–3% rate of metastatic spread in level V supporting the notion of sparing posterior triangle while performing neck dissection [10, 11]. It was also concluded that level V dissection can be avoided even if suspicious lymph nodes are encountered at level IV [3]. In the current study, a marked difference in regional control was observed between node negative and node positive patients who underwent neck dissection. Level I–IV SND was performed more frequently (82% versus 18%). Overall, 8% of total patients who underwent level I–IV SND had level IV involvement. Tongue was the most common site of primary and level II A or subdigastric level was the most common involved sublevel. Overall 22 patients failed regionally with a high preponderance of ipsilateral failures (18 versus 4). Regional failure was more common in the first year after surgery and highlights importance of meticulous surveillance in this time period. Limitations of the current study include its retrospective design and missing data. Sublevels of regional failures in neck could not be determined as majority of patients had huge fixed neck recurrences involving more than one sublevels of neck. In addition the prognostic role of extracapsular extension could not be determined due to small number of patients who had extracapsular involvement.

There are no set guidelines for management of clinically node negative neck in early oral cancer. Studies have been reported both in favor of elective neck dissection and wait and see policy [12–14]. Current guidelines recommend elective neck dissection when probability of occult metastasis is 20% [2]. Recently a meta-analysis showed elective neck dissection to be a better option [15]. A high occult metastatic rate of 37%, low socioeconomic status, and distant geographic location of our patients made elective neck dissection a more suitable option in our setting. In critical assessment of supraomohyoid neck dissection, removal of level I–III was found appropriate for staging of cN0 patients. Occult metastasis was present in 31% out of a total of one hundred and fifteen patients included in the study [16]. In the current study, a high proportion of cN0 patients underwent level I–IV neck dissection but only 14 patients were found to have pathological evidence of disease in level IV. Since the results on the findings of the current study, practice has already been modified and level IV dissection is only performed in patients with clinical nodal disease in level III/IV in neck in our institute now.

Radiotherapy either in pre- or postoperative setting has shown its benefit with reduction in the incidence of neck failures by 50% irrespective of the N stage [17–19]. The choice of pre- or postoperative radiation remains largely institutional with surgeons preferring PORT as it reduces the operative complications and makes performance of neck dissection relatively easy [20]. Adjuvant radiotherapy has also been recommended in clinically node negative contralateral neck to reduce the rate of contralateral neck failure [21]. Two trials conducted in Europe (European Organization Research and Treatment of Cancer (EORTC)) and the United States (Radiation Therapy Oncology Group (RTOG)) have shown better locoregional control in patients with extracapsular spread and/or positive surgical margins who received postoperative chemoradiation [22, 23]. Chemoradiation has been advocated in presence of poor prognostic factors like stage III–IV disease, perineural infiltration, vascular embolisms, and/or clinically enlarged level IV–V lymph nodes [14]. In the current study, 88% patients received radiation of which 23% were in the setting of concurrent chemoradiotherapy. Tumor grade, extracapsular spread, lymphovascular invasion, and pathological N stage were significant variables for regional control in present study. However the number of patients with these variables was very small.

Radical neck dissection (RND) remained the procedure of choice in node positive patients for greater part of the 20th century. Strong [24] in their study showed a regional recurrence rate of 54.3% in node positive patients and 71.3% in patients with positive nodes at multiple levels. This leads to several questions regarding the oncological benefit and morbidity associated RND. In the past two decades the use of SND has gained popularity in the management of node positive patients partly because of the comparable regional control rate in patients with occult disease undergoing elective neck dissection and also due to an increasing use of PORT for better disease control. Andersen et al. [3] in their study of 106 patients with 129 therapeutic SND had 9 regional failures. The study included all sites of head and neck region and >50% patients had N1 disease. In another study on effectiveness of SND in clinically node positive neck including all primary sites of head and neck region, 54 patients underwent SND including 33 patients with pN2/3 disease. There were 2 ipsilateral recurrences and SND showed better disease control but did not reach statistical significance [25].

With an increasing trend of performing limited and site specific lymphadenectomy procedures, a comprehensive classification of neck dissection has been proposed that is logical, simple, and easy to remember [26]. In the current study, the authors have compared the old nomenclature with the proposed classification and found the new classification better at defining precisely the lymphatic and non-lymphatic structures excised.

The role of induction chemotherapy in head and neck squamous cell carcinoma before a definitive surgical intervention is not well defined [27]. In the current study, 45 patients received induction chemotherapy. On histopathology, only 1 patient had T4 tumor out of 6 patients with cT4 tumors who received induction chemotherapy. Out of 23 patients with cT3 disease who received induction chemotherapy, only 3 had pT3 disease. This favors the role of induction chemotherapy in downstaging of head and neck tumors before surgical resection; however conclusions cannot be drawn due to heterogeneous nature of our cohort.

We also made an effort to identify a subgroup of patients in which pathological nodal positivity was absent. This could potentially represent a group in which SND could be avoided. Almost 90% patients with well-differentiated, T1 tumors and cN0 neck did not have pathological nodal disease after SND. This group could potentially represent a subgroup that might benefit from wait and see policy under close surveillance. Further studies are needed to address this issue taking into consideration several other prognostic variables.

The current study reports regional failures after SND in previously untreated squamous cell carcinoma of oral cavity in both node negative and positive patients. It highlights several important issues. Induction chemotherapy may have a beneficial role in locally advanced head and neck squamous cell carcinomas but this needs to be confirmed in future trials. There might be a subgroup of patients with well-differentiated, clinically node negative T1 tumors that can be safely managed with observation alone. As the role of neck dissection becomes more conservative with ever-expanding application of PORT and CRT providing improved regional control, application of level I–IV SND should be limited. Superselective neck dissection might become the standard for regional management of neck in oral squamous cell carcinoma.

## Figures and Tables

**Table 1:** Patient characteristics and treatment modalities in clinically node negative and positive patients.

	Node negative patients		Node positive patients		
	Number (*N*)	Percent (%)	Number (*N*)	Percent (%)	
Gender					Not significant
Male	104	66	38	62	
Female	54	34	23	38	
Subsite					0.009
Anterior tongue	119	75	40	66	
Upper alveolus	1	1	0	0	
Lower alveolus	22	14	10	16	
Buccal mucosa	16	10	6	10	
Retromolar trigone	0	0	2	3	
Lips	0	0	3	5	
Grade					Not significant
Well	79	50	31	51	
Moderate	64	40	21	34	
Poor	13	10	9	15	
Treatment modality					0.0001
S	25	16	1	2	
S + RT	91	58	30	49	
S + CRT	16	10	11	18	
C + S + RT	15	10	6	10	
C + S + CRT	11	7	13	21	
Level					0.002
I–III	33	21	2	3	
I–IV	125	79	59	97	

S: surgery, RT: radiation therapy, and C: chemotherapy.

**Table 2:** Clinical and pathological staging of patients according to AJCC guidelines.

Stage	Clinical number (*N*)	Percent (%)	Pathological number (*N*)	Percent (%)
T stage				
T0	—	—	8	4
T1	65	29	97	44
T2	98	45	81	37
T3	28	13	13	6
T4	28	13	20	9
N Stage				
N0	158	72	114	52
N1	34	16	32	14
N2a	6	3	0	0
N2b	16	7	67	31
N2c	5	2	5	2
N3	0	—	1	1
Overall stage				
0	—		6	3
I	53	24	55	25
II	70	32	38	17
III	46	21	32	15
IV	50	23	88	40

**Table 3:** Comparison of old and proposed classification.

Selective neck dissection			Proposed classification		
	Number (%)	Percent (%)		Number	Percent (%)
Level I–III	35 (16)	16	ND (I–III)	35	16
Level I–IV	184 (84)	84	ND (I–IV)	151	69
			ND (I–IV, IJV, CN XI)	2	1
			ND (I–IV, IJV)	29	13
			ND (I–IV, CN XI)	2	1

ND: neck dissection, IJV: internal jugular vein, and CN XI: accessory nerve.

**Table 4:** Characteristics of patient who received induction chemotherapy.

	Number (*N* = 45)	Percent (%)
Gender		
Male	30	66
Female	15	34
Clinical T stage		
T1	1	2
T2	15	33
T3	23	51
T4	6	14
Clinical N stage		
N0	26	58
N+	19	42
Grade		
Well	21	47
Moderate	17	38
Poor	7	15
Pathological T stage		
pT0	6	14
pT1	25	55
pT2	10	22
pT3	3	7
pT4	1	2
Pathological N stage		
N0	19	42
N+	26	58

**Table 5:** Regional failures in clinically node negative and node positive neck.

	Node negative		Node positive		Total
	*N* = 14		N = 8		
	Number	Percent	Number	Percent	
Tumor size					
T1	5	36	2	33	7
T2	6	43	4	43	10
T3	3	21	0	8	3
T4	0	0	2	16	2
Site					
Ipsilateral	11	80	7	86	18
Contralateral	3	20	1	14	4
Primary					
Tongue	10	72	6	75	16
Lower alveolus	1	7	1	12.5	2
Buccal mucosa	2	14	1	12.5	3
Upper alveolus	1	7	0	0	1
Extracapsular					
Present	2	50	1	33	3

**Table 6:** Prognostic variables for 5-year regional control.

Prognostic factor	Number (*n*)	5-year regional control (%)	*P* value
Tumor grade			
Well	110	84	0.006
Mod	84	87	
Poorly	25	62	
Lymphovascular invasion			
Positive	12	68	0.044
Negative	153	85	
Unknown	54	85	
Perineural invasion			
Positive	28	75	Not significant
Negative	140	84	
Unknown	51	84	
Pathological N stage			
N0	114	95	<0.0001
N+	105	81	
Pathological T stage			
T1-T2	186	82	Not significant
T3-T4	33	91	
Number of positive nodes			
1, 2	57	70	Not significant
3, 4, 5	26	71	
>5	22	61	

**Table 7:** Frequency of node positivity on histopathology in patients with well-differentiated tumors and clinically node negative neck.

	pN0	%	pN1	%	pN2a	%	pN2b	%	Total
cT1	27	87	2	6.5	0	—	2	6.5	31
cT2	17	53	7	22	0		8	25	32
cT3	5	71	0	—	1	14.5	1	14.5	7
cT4	6	67	1	11	0	—	2	22	9
	55	70	10	13	1	1	13	16	79

p: pathological.

c: clinical.
